# RETRACTION: α‐Synuclein Conformational Antibodies Fused to Penetratin are Effective in Models of Lewy Body Disease

**DOI:** 10.1002/acn3.70270

**Published:** 2025-11-30

## Abstract

**RETRACTION**: SpencerB. , WilliamsS. , RockensteinE. , ValeraE. , XinW. , ManteM. , FlorioJ. , AdameA. , MasliahE. , and SierksM.R. , “α‐Synuclein Conformational Antibodies Fused to Penetratin are Effective in Models of Lewy Body Disease,” Annals of Clinical and Translational Neurology 3, no. 8 (2016): 588–606, 10.1002/acn3.321.27606342 PMC4999592

The above article, published online on 16 June 2016, in Wiley Online Library (http://onlinelibrary.wiley.com/), has been retracted by agreement between the journal Editor‐in‐Chief, Ahmet Hoke; the American Neurological Association; and Wiley Periodicals LLC. Following publication, a third party contacted the publisher with concerns about duplications in Figures 1A, 3A, 3C, and 4A. Additionally, duplicated panels were discovered for Figure 5 with an earlier publication by some of the same authors (Fields et al., 2016 [https://doi.org/10.1186/s12974‐016‐0585‐8]). The retraction has been agreed to because of evidence that significant portions of multiple figures were duplicated, affecting the interpretation of the data and results presented. Author Eliezer Masliah did not indicate his agreement with the retraction. The other authors did not respond to communications from the Publisher regarding the retraction.

**RETRACTION**: B. Spencer , S. Williams , E. Rockenstein , E. Valera , W. Xin , M. Mante , J. Florio , A. Adame , E. Masliah , and M.R. Sierks , “α‐Synuclein Conformational Antibodies Fused to Penetratin are Effective in Models of Lewy Body Disease,” Annals of Clinical and Translational Neurology 3, no. 8 (2016): 588–606, 10.1002/acn3.321.27606342 PMC4999592

The above article, published online on 16 June 2016, in Wiley Online Library (http://onlinelibrary.wiley.com/), has been retracted by agreement between the journal Editor‐in‐Chief, Ahmet Hoke; the American Neurological Association; and Wiley Periodicals LLC. Following publication, a third party contacted the publisher with concerns about duplications in Figures 1A, 3A, 3C, and 4A. Additionally, duplicated panels were discovered for Figure 5 with an earlier publication by some of the same authors (Fields et al., 2016 [https://doi.org/10.1186/s12974‐016‐0585‐8]). The retraction has been agreed to because of evidence that significant portions of multiple figures were duplicated, affecting the interpretation of the data and results presented. Author Eliezer Masliah did not indicate his agreement with the retraction. The other authors did not respond to communications from the Publisher regarding the retraction.

